# Inflammatory phenotypes after total knee arthroplasty: a testable framework for biomarker-stratified enhanced recovery and precision rehabilitation

**DOI:** 10.3389/fmed.2026.1938901

**Published:** 2026-09-11

**Authors:** Xu Chen, Xiaoling Luo, Yaxi Qiu, Qin Zhou, Shangjie Zhong, Hongyan Zhu, Mei Yao, Peifang Li

**Affiliations:** Department of Orthopedic Surgery, West China Hospital, Sichuan University/West China School of Nursing, Sichuan University, Chengdu, China

**Keywords:** arthrogenic muscle inhibition, biomarker stratification, C-reactive protein, enhanced recovery after surgery, inflammatory phenotype, interleukin-6, precision rehabilitation, total knee arthroplasty

## Abstract

Recovery after total knee arthroplasty (TKA) remains heterogeneous despite mature surgical techniques and standardized enhanced recovery after surgery (ERAS) pathways. Perioperative inflammation offers an unusually actionable basis for stratification because it can be measured before surgery, tracked during recovery, linked to pain, swelling, and quadriceps dysfunction, and modified by available interventions. We advance a three-axis inflammatory phenotype comprising preoperative inflammatory tone, acute response magnitude, and resolution kinetics. Preoperative systemic immune-inflammation index (SII), conventionally calculated from a CBC obtained at a prespecified time point, can contribute to Axis 1, and serial SII may be evaluated as a candidate trajectory; the modified frailty index (mFI) summarizes preoperative deficit burden. The framework tests whether their information is complemented by the joint temporal response to surgical perturbation and the coordination of systemic, local, and functional recovery. The three axes are primarily continuous dimensions; any latent classes are exploratory and require reproducibility across sites. Serial interleukin-6, C-reactive protein, fibrinogen, cytokine-panel, swelling, and temperature studies support these complementary dimensions. Prognostic cohorts connect inflammatory profiles with pain, function, complications, and recovery trajectories, while randomized trials demonstrate that the acute response can be pharmacologically shifted. The framework integrates accessible C-reactive protein and complete blood count-derived ratios with targeted cytokines and local swelling or temperature. Each signal is paired with mechanism-aligned outcomes such as voluntary activation, quadriceps strength, Timed Up and Go performance, and Knee injury and Osteoarthritis Outcome Score domains. The model organizes prospective, safety-gated tests of phenotype-adapted ERAS and rehabilitation, including protocol- or trial-based anti-inflammatory strategies, compression, neuromuscular electrical stimulation, nutritional support, progressive resistance training, and extended supervised rehabilitation. Five prospective predictions link each phenotype axis to a distinct recovery mechanism and intervention response. Multicenter trajectory cohorts can derive and validate the phenotype, followed by biomarker-stratified factorial trials powered for treatment-by-phenotype interaction. This framework converts perioperative inflammation from an isolated laboratory signal into a dynamic recovery phenotype and proposes a testable blueprint for prospective evaluation of adaptive ERAS and precision rehabilitation after TKA. Incremental utility relative to existing indices remains a prospective hypothesis.

## 1 Introduction

Total knee arthroplasty (TKA) reliably relieves pain and restores mobility in end-stage knee osteoarthritis. A substantial subgroup nevertheless experiences persistent pain, dissatisfaction, or incomplete functional recovery. In a population study of 1,703 primary TKAs, 19% of patients were dissatisfied, and broader syntheses estimate that long-term pain affects a clinically important minority (1–3). Early quadriceps weakness is even more common and directly constrains transfers, walking, and stair negotiation. These outcomes define the central precision-recovery problem: technically successful surgery can produce markedly different biological and functional trajectories.

Enhanced recovery after surgery (ERAS) pathways have improved perioperative reliability through multimodal analgesia, blood-conservation strategies, early mobilization, and coordinated discharge planning (4). Their success creates a strong platform for personalization. Standardized core care controls avoidable variation, while targeted adaptation can address biological variation that remains after the pathway is implemented. The next advance is therefore an adaptive ERAS model that preserves a common evidence-based foundation and intensifies selected components for patients with identifiable recovery mechanisms.

Inflammation is especially well suited to this role. It is observable before incision, changes rapidly after surgical trauma, and remains connected to local tissue responses during rehabilitation. Serum and synovial markers, complete blood count-derived ratios, limb swelling, effusion, and peri-patellar temperature provide complementary views of the response. A systematic review of biomarker-guided TKA intervention studies found frequent concordance between biomarker suppression and improvement in at least one early clinical outcome (5). The review also revealed a diverse evidence base that can be reorganized around mechanism and timing. Recent reviews further demonstrate that interleukins, plasminogen activator inhibitor-1, C-reactive protein (CRP), and tumor necrosis factor participate in an interconnected perioperative response (6).

We propose that perioperative inflammation forms a dynamic recovery phenotype with three axes: preoperative inflammatory tone, acute response magnitude, and resolution kinetics. The phenotype is designed to support decisions. Each axis maps to a distinct measurement window, biological interpretation, rehabilitation mechanism, and trial hypothesis. The framework aligns directly with precision medicine because it links patient-level molecular and digital signals to tailored treatment selection and recovery monitoring. Preoperative SII, as conventionally calculated from a CBC obtained at a prespecified time point, can contribute to Axis 1; serial SII may also be evaluated as a candidate trajectory. The mFI summarizes preoperative deficit burden. Neither conventional implementation by itself encodes the full joint perturbation-response-resolution pattern or systemic-local-functional discordance. The proposed incremental value is therefore a prospective hypothesis rather than an established advantage (7–9).

## 2 Evidence synthesis approach

We conducted a targeted, non-systematic evidence synthesis for hypothesis development using PubMed searches updated through 18 August 2026. Search concepts combined TKA with inflammatory biomarkers or cytokines, complete blood count-derived indices, swelling or temperature, arthrogenic muscle inhibition, pain sensitization, glucocorticoids or other anti-inflammatory interventions, rehabilitation, ERAS, prediction modeling, and digital monitoring. We prioritized human longitudinal cohorts, randomized trials, systematic reviews, and methodological guidance that informed reference kinetics, candidate measurement, mechanisms, intervention modifiability, prediction, implementation, or trial design. Because this Hypothesis and Theory article was not designed as a systematic review, study selection was purposive rather than exhaustive, no pooled effect estimate or formal risk-of-bias appraisal was undertaken, and the cited evidence should not be interpreted as a complete efficacy review. The synthesis is used to generate testable propositions and exposes heterogeneity and null findings alongside positive studies.

## 3 Central hypothesis: a three-axis inflammatory recovery phenotype

The central hypothesis is that clinically meaningful recovery heterogeneity after TKA can be captured by the interaction of inflammatory tone, response magnitude, and resolution kinetics. These axes describe a biological process from baseline susceptibility through surgical perturbation to recovery. Their joint pattern should carry more actionable information than a single postoperative laboratory value.

### 3.1 Axis 1: preoperative inflammatory tone

Preoperative inflammatory tone represents the biological context in which surgical stress occurs. Contributors include osteoarthritis synovitis, adiposity, metabolic disease, immune ageing, pain sensitization, and low-grade systemic inflammation. Synovial-fluid TNF-alpha, MMP-13, and IL-6 predicted less pain improvement two years after TKA in a small prognostic cohort (10). A larger serum-profiling study identified inflammatory clusters associated with 12-month pain and function (11). In routine-care data, neutrophil-, monocyte-, and platelet-to-lymphocyte ratios and the systemic immune-inflammation index predicted aggregate complications after total joint arthroplasty (7). TKA-specific analyses have also linked the systemic inflammatory response index and systemic immune-inflammation index with clinical outcomes (8).

This axis can begin with accessible measures. CRP, neutrophil-to-lymphocyte ratio, monocyte-to-lymphocyte ratio, platelet-to-lymphocyte ratio, and systemic immune-inflammation index are available from standard laboratory testing. Targeted serum or synovial cytokines can refine the phenotype in research cohorts. Preoperative IL-6 and leptin have been associated with osteoarthritis pain severity, illustrating how systemic and metabolic signals can identify a biologically distinct starting state (12). The trait axis is therefore positioned as a low-burden first screen that can guide deeper profiling.

### 3.2 Axis 2: acute response magnitude

Acute response magnitude describes the amplitude, slope, and cumulative exposure generated by surgery. Serial studies establish reproducible patterns while demonstrating protocol sensitivity. Under one ERAS pathway, IL-6 peaked at 48 h and CRP at postoperative day 3, with recovery toward baseline by two weeks (13). In another cohort, IL-6 peaked at 12 h and CRP at 48 h (14). IL-6 changed more rapidly than CRP in a mixed hip-knee cohort, and area under the curve (AUC) efficiently summarized repeated measurements (15). Fibrinogen and albumin-based ratios extended the measurable window beyond CRP (16, 17).

Additional studies broaden the response signal. Circulating cytokines rise after hip and knee arthroplasty (18), and combined IL-6, CRP, and skin-temperature measurement provides a bridge between molecular and local recovery signals (19). CRP kinetics also differ by arthroplasty type, reinforcing the value of procedure-specific reference curves (20). Acute response magnitude can therefore be quantified through peak, slope, AUC, and deviation from a protocol-specific expected trajectory.

### 3.3 Axis 3: resolution kinetics

Resolution kinetics captures the rate at which systemic and local signals return toward the patient's reference state. This axis is clinically attractive because rehabilitation is already underway while inflammation, swelling, temperature, and muscle activation are changing. Early postoperative swelling is influenced by obesity and preoperative joint pain (21). Bioimpedance and serial circumference studies connect swelling with quadriceps weakness, gait speed, and Timed Up and Go (TUG) performance (22, 23). Persistent pain cohorts further associate synovitis, effusion, sensitization, and inflammatory mediators with later pain and function (24, 25).

**Figure 1:**
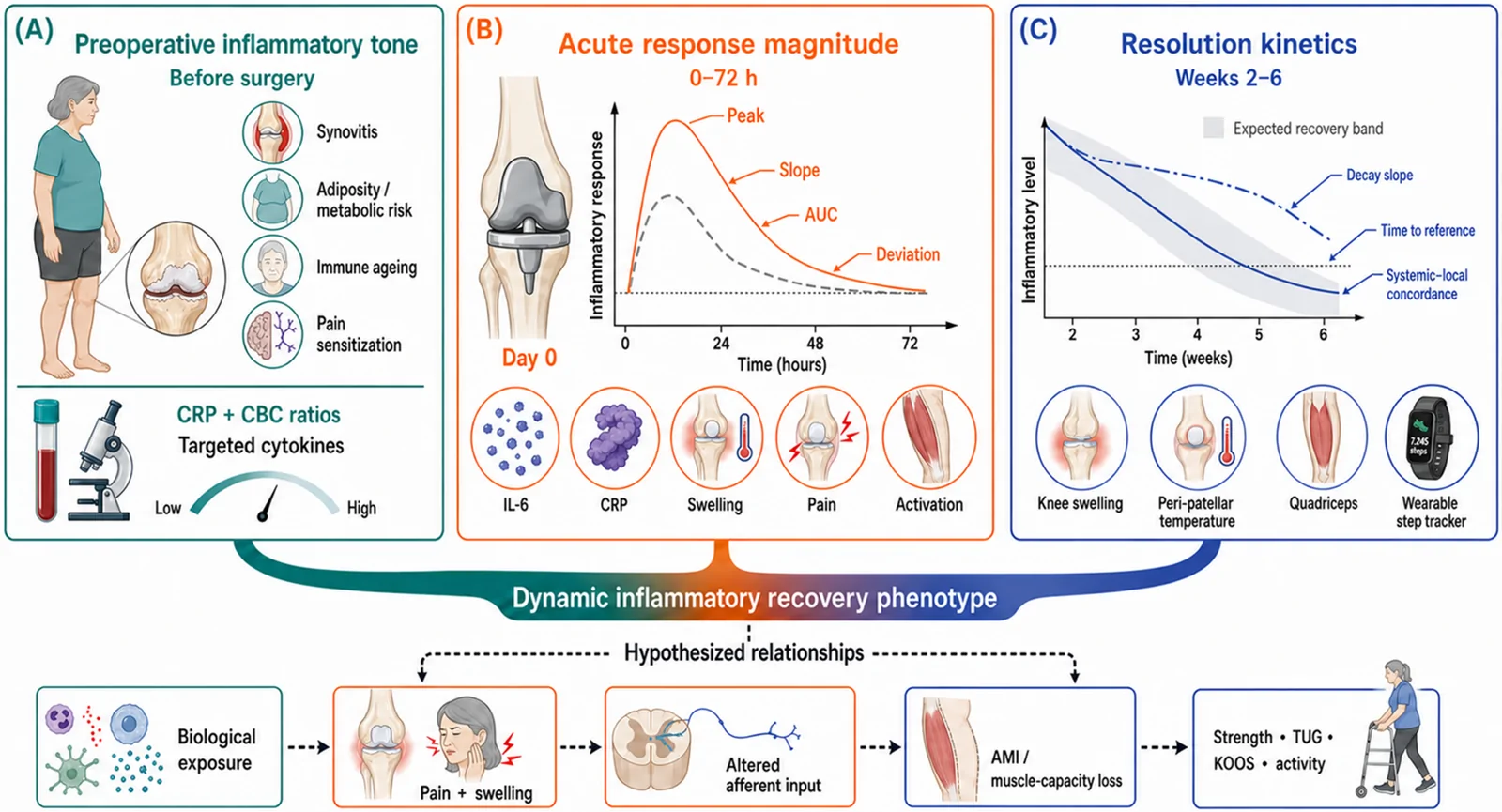
Three-axis inflammatory recovery phenotype after total knee arthroplasty. The conceptual framework integrates three complementary dimensions of perioperative recovery: **(A)** preoperative inflammatory tone, measured before surgery as the biological context in which surgical stress occurs; **(B)** acute response magnitude, characterized by peak, slope, area under the curve, and deviation from an expected trajectory during the first 72 h; and **(C)** resolution kinetics, defined by the rate and coordination with which systemic inflammation, local joint responses, neuromuscular function, and mobility return toward an expected recovery trajectory during weeks 2–6. Candidate measures include C-reactive protein (CRP), complete blood count-derived inflammatory ratios, interleukin-6 (IL-6), fibrinogen-based measures, swelling or effusion, peri-patellar temperature, pain during movement, voluntary activation, quadriceps strength, Timed Up and Go (TUG), Knee injury and Osteoarthritis Outcome Score (KOOS), and free-living activity. The joint pattern is proposed to define a dynamic inflammatory recovery phenotype linking biological exposure to pain, swelling, arthrogenic muscle inhibition, muscle-capacity loss, and functional recovery. Arrows indicate hypothesized directional relationships for prospective testing and do not imply established causality. AUC, area under the curve; CBC, complete blood count; CRP, C-reactive protein; IL-6, interleukin-6; KOOS, Knee injury and Osteoarthritis Outcome Score; TUG, Timed Up and Go. Wearable mobility and continuous peri-patellar temperature are optional enhanced research modules, not requirements of the minimum viable implementation dataset. Temperature and mobility are shown as separate candidate signals; any derived thermo-mobility coupling index remains exploratory and requires external validation.

Local and digital measures can make resolution observable outside the laboratory. Swelling assessment methods have been systematically compared (26), and a recent review organizes interventions that reduce swelling after TKA (27). In a single-center prospective cohort of 142 patients, continuous peri-patellar temperature and accelerometry were combined into derived thermo-mobility coupling indices. Coupling during postoperative weeks 2–4 was associated with 12-week functional outcomes after covariate adjustment, but independent external validation remains necessary (28). This limited evidence supports treating thermo-mobility coupling as an optional exploratory feature within the enhanced module, not as an established clinical marker. The proposed relationship among preoperative inflammatory tone, acute response magnitude, resolution kinetics, and mechanism-aligned recovery outcomes is summarized in Figure 1. The study-level evidence supporting the three axes is synthesized in Table 1, while Table 2 specifies the minimum dataset, sampling windows, derived features, outcomes, standardization requirements, and validation targets for each axis. Table 2 separates a true minimum viable implementation dataset from optional enhanced research measurements.

**Table 1:** Study-level evidence matrix supporting inflammatory recovery phenotyping after TKA.

Study (reference)	Design and sample	Phenotype axis	Measures and timing	Principal finding	Contribution to the framework	Main limitation
Huang et al. (13)	Prospective ERAS cohort; 100 primary TKA	Acute magnitude and resolution	IL-6 and CRP at baseline, 12 h, 48 h, day 3, and week 2	IL-6 peaked at 48 h and CRP at day 3; both approached baseline or normal range by week 2	Supplies an ERAS-specific reference trajectory and candidate deviation windows	Single-center trajectory; not designed to establish prognostic or infection thresholds
Maniar et al. (14)	Prospective diagnostic cohort; 50 uncomplicated primary TKA	Acute magnitude and resolution	IL-6 and CRP at baseline, 12 h, 48 h, day 4, and week 2	IL-6 peaked at 12 h and normalized by week 2; CRP peaked at 48 h and remained above baseline at week 2	Demonstrates that marker-specific peak and recovery times should not be collapsed into one postoperative value	Single surgeon and standardized protocol limit transportability
Wasko et al. (15)	Prospective arthroplasty cohort; 100 hip or knee procedures	Acute magnitude	CRP, IL-1beta, IL-6, IL-8, and NT-proCNP over the first 5 postoperative days	IL-6 had faster kinetics than CRP, was less influenced by weight, and was efficiently summarized by AUC	Supports IL-6 and AUC as high-information measures of surgical inflammatory exposure	Mixed hip and knee cohort; early window only
Oelsner et al. (16)	Retrospective healthy total-joint cohort; 180 procedures	Preoperative tone and resolution	CRP and fibrinogen preoperatively, perioperatively, and at weeks 2 and 6	CRP returned to baseline by week 2 and fibrinogen by week 6; higher baseline CRP predicted a larger acute-phase response	Links baseline inflammatory tone to response magnitude and separates fast from slow resolution markers	Mixed arthroplasty population selected for low comorbidity
Amaro et al. (17)	Retrospective primary TKA cohort	Acute magnitude and resolution	Albumin, CRP, fibrinogen, and fibrinogen-to-albumin ratio through week 6	Albumin fell on day 1 and largely recovered by week 2; fibrinogen-to-albumin ratio peaked later and normalized by week 6	Adds inexpensive negative and composite acute-phase reactants for later resolution assessment	Retrospective design; clinical decision thresholds were not validated
Gandhi et al. (10)	Prognostic cohort; 28 TKA	Preoperative tone	Serum and synovial cytokines at surgery; WOMAC pain at 2 years	Higher synovial TNF-alpha, MMP-13, and IL-6 independently predicted less pain improvement	Provides direct evidence that a preoperative local inflammatory profile may identify persistent-pain susceptibility	Very small cohort and multiple biomarker comparisons
Giordano et al. (11)	Secondary analysis; 127 TKA candidates and 39 healthy controls	Preoperative tone	Multiprotein serum panel before surgery; VAS and KOOS at 12 months	Unsupervised clustering identified two inflammatory subgroups with different 12-month pain and function	Supports multivariable inflammatory endotypes rather than isolated-marker stratification	Exploratory clustering without external validation or a locked classifier
Jones et al. (7)	Retrospective database study; 20,061 TKA within 32,868 arthroplasties	Preoperative tone and risk	Preoperative NLR, MLR, PLR, and SII; complications and length of stay	Higher CBC-derived ratios were associated with complications; candidate TKA cut points included NLR 3.7, MLR 0.41, PLR 205.1, and SII 1,013.1	Identifies scalable screening variables for broad implementation cohorts	Administrative data, outcome-oriented thresholds, and no direct recovery-mechanism measures
Yang et al. (21)	Prospective observational cohort; 110 TKA	Acute local response	Limb circumference on postoperative days 1–5, pain, BMI, CRP, and blood counts	Proximal swelling peaked on day 3; BMI and site-specific pain were stronger predictors than measured inflammatory markers	Shows that local swelling phenotypes require clinical and anthropometric context	Early single-center window and circumference-based swelling measurement
Giordano et al. (24)	Exploratory cross-sectional follow-up; 76 patients at 5 years after TKA	Persistent inflammatory state	Forty-four plasma markers, VAS, pain catastrophizing, and Oxford Knee Score	Distinct markers were associated with high pain, catastrophizing, or poor function, consistent with low-grade inflammation in a subset	Extends phenotyping beyond the perioperative window to persistent symptoms	Biomarkers measured only at 5 years; temporality and causality cannot be established
Si et al. (34)	Prospective cohort; 96 TKA, with synovial samples in 54	Acute magnitude and pain coupling	Serum cytokines and muscle-damage markers at baseline and postoperative days 1, 2, 3, and 5	Serum PGE2, IL-6, CRP, CK, myoglobin, and LDH correlated with acute pain at selected time points	Links biological response to activity-related pain and supports time-matched outcomes	Correlational analysis with multiple testing and no treatment-response evaluation
Pua (22)	Longitudinal cohort; 85 unilateral TKA	Local response and resolution	Bioimpedance swelling on days 1, 4, 14, and 90; strength and fast gait speed	Swelling increased about 35% on day 1, remained about 11% above baseline on day 90, and was associated with weakness and slower gait	Establishes swelling as a persistent, functionally relevant local phenotype feature	Observational association; systemic biomarkers were not integrated
Loyd et al. (23)	Prospective repeated-measures cohort; 53 TKA	Local response magnitude	Bioimpedance peak and cumulative swelling; quadriceps strength and TUG at weeks 2 and 6	Peak swelling, rather than cumulative swelling, predicted TUG and contributed to 6-week strength variance	Prioritizes peak swelling as a parsimonious early feature for rehabilitation stratification	Modest sample and limited covariate control
Loyd et al. (35)	Multicenter observational study; 53 TKA	Nociception-activation coupling	Knee and forearm pressure-pain thresholds, voluntary activation, and quadriceps strength at baseline and week 6	Local pressure-pain threshold was associated with activation and strength; activation mediated part of the strength relationship	Defines a plausible pathway from local nociception to arthrogenic muscle inhibition	Mechanistic association without inflammatory biomarker measurement or intervention
Kurien et al. (25)	Exploratory cohort; 46 patients at 6 months after TKA	Delayed local resolution and persistent pain	MRI synovitis and effusion, quantitative sensory testing, pain, and catastrophizing	Synovitis, effusion, temporal summation, and catastrophizing independently contributed to pain intensity	Shows that delayed-resolution phenotypes may combine local inflammation and central pain amplification	Small cohort; findings require prospective confirmation
Carr et al. (28)	Prospective single-center cohort; 142 primary TKA	Resolution kinetics and cross-domain coupling	Continuous peri-patellar temperature patch and thigh-mounted accelerometer for 6 weeks; coupling during weeks 2–4; outcomes at 12 weeks	Higher maladaptive coupling was associated with worse KOOS-ADL, reduced flexion, slower TUG, and delayed opioid cessation after covariate adjustment	Provides direct proof-of-concept for integrating local thermal and free-living mobility trajectories as an exploratory Axis 3 feature	Single-center observational associations; no independent external or long-term validation; causality, thresholds, and clinical utility remain unestablished
Kürüm et al. (8)	Retrospective cohort; 187 TKA	Complication-sensitive inflammatory deviation	Preoperative and postoperative SIRI, SII, NLR, and PLR; periprosthetic joint infection	SIRI and SII were higher with infection, but proposed postoperative thresholds had limited specificity	Reinforces that phenotype monitoring must include a complication-detection safety pathway	Retrospective diagnostic analysis; thresholds should not be used alone
Rockel et al. (36)	Multiomic endotyping study; 414 knee osteoarthritis patients	Preoperative systems phenotype	Plasma, synovial-fluid, and urine microRNA/metabolomic data plus clinical variables	Multimodal clustering produced three molecular endotypes; integrated molecular and clinical data improved post-TKA response classification	Demonstrates a discovery route for biologically distinct endotypes that can be mapped onto the three clinical axes	High-dimensional model requires external validation and simplified clinical translation
Harmelink et al. (37)	Prospective trajectory cohort; 218 TKA	Functional resolution phenotype	TUG, KOOS-ADL, and pain at baseline, day 3, weeks 2 and 6, and 1 year	Early latent recovery classes were distinguishable and associated with 1-year outcomes	Provides outcome trajectories against which biomarker and local-response trajectories can be tested	Selected high-intensity physiotherapy population and very small poor-recovery classes

**Table 2:** Operational specification of the three-axis inflammatory recovery phenotype: minimum viable and enhanced research datasets.

Axis	Biological construct	Nested measurement strategy	Recommended sampling window	Derived features	Mechanism-aligned outcomes	Required standardization and covariates	Decision and validation target
Preoperative inflammatory tone	Stable or slowly varying systemic, metabolic, and joint inflammatory susceptibility before surgical stress	**MVD:** routine CBC-derived ratios; CRP if already available; BMI or diabetes; activity pain; simple function or strength. **Enhanced:** repeat preoperative CBC or CRP; targeted serum or synovial cytokines.	**MVD:** routine preoperative visit; repeat only when clinically indicated or feasible. **Enhanced:** prespecified repeat baseline to assess short-term reliability.	Standardized continuous trait score; marker-specific z scores; metabolic-inflammatory interaction terms	Baseline activation and strength; early swelling; pain and opioid need; complications; 6–12-week KOOS and TUG	Intercurrent infection, inflammatory disease, corticosteroid or immunomodulator use, obesity, diabetes, anemia, smoking, assay platform, and timing	Test incremental prediction beyond time-matched SII (preoperative or serial), mFI, and conventional risk factors and treatment interaction with prehabilitation or anti-inflammatory strategies; do not adopt fixed cut points before external validation.
Acute response magnitude	Amplitude and cumulative systemic and local exposure generated by surgery	**MVD:** one in-hospital or discharge checkpoint aligned with routine care; wound or symptom review; activity pain; standardized swelling; simple mobility or activation; CBC or CRP only if clinically obtained. **Enhanced:** serial IL-6 or CRP; peri-patellar temperature; detailed effusion or activation measures.	**MVD:** one routine in-hospital or discharge checkpoint. **Enhanced:** baseline plus 12–24 h and 48–72 h; optional day 5–7.	**MVD:** simple change from baseline or deviation at one checkpoint. **Enhanced:** peak, slope, AUC, time to peak, and deviation from a protocol-specific reference curve.	Pain during mobilization; opioid consumption; activation; quadriceps strength; walking distance; TUG; discharge readiness	Glucocorticoid dose and timing, anesthesia and analgesia, tourniquet, blood loss, transfusion, operative duration, surgical technique, cryotherapy/compression, assay timing, and infection screen	Determine whether an exaggerated response predicts differential benefit from anti-inflammatory dosing or AMI-focused rehabilitation; verify target engagement separately from functional benefit
Resolution kinetics	Rate and coordination with which systemic inflammation, local joint response, pain, and function return toward an expected trajectory	**MVD:** week-2 phone or clinic wound and symptom review, activity pain, swelling, mobility, and adherence; week-6 routine KOOS and TUG or equivalent. No mandatory wearable or post-discharge research phlebotomy. **Enhanced:** serial CRP or fibrinogen, imaging, continuous temperature, and wearable mobility.	**MVD:** week 2 and week 6 using routine remote or clinic contacts.@**Enhanced:** optional daily remote monitoring in weeks 1–6 and later symptom-focused assessment.	**MVD:** residual symptoms or function and change between routine checkpoints.@**Enhanced:** half-life or decay slope, time to reference, candidate thermo-mobility coupling indices (exploratory; external validation required), multivariate trajectory class, and systemic-local discordance.	Persistent pain; KOOS domains; TUG; gait speed; activity recovery; return to participation; complication detection	Wound status, infection or thrombosis evaluation, rehabilitation dose and adherence, activity exposure, analgesic use, sleep, psychosocial factors, and missing repeated measures	Test whether delayed or discordant resolution identifies benefit from mechanism-focused escalation after safety triage; prespecify, lock, and validate MVD and enhanced models separately.

The minimum viable dataset (MVD) is a lower-burden screening and checkpoint-management dataset. It cannot reliably estimate full IL-6 or CRP AUC, biomarker half-life, or latent trajectory classes. MVD and enhanced models must be prespecified, locked, and validated separately; structural device or sampling unavailability must not be treated as ordinary random missingness.

### 3.4 Construct definition, novelty, and reproducibility

In this framework, a marker is an individual measurement, an axis is a continuous dimension derived from prespecified longitudinal features, and the inflammatory recovery phenotype is the joint multivariate pattern across the three axes. The primary representation is continuous: baseline level, peak or AUC, slopes, time to recovery, and systemic-local-functional discordance retain ordering and avoid premature cut points. Latent-class or trajectory-based groupings are exploratory simplifications, not assumed biological entities; they will be retained only if statistically stable, clinically interpretable, adequately sized, and reproducible in independent sites (29, 30).

The construct is complementary to, rather than a replacement for, existing indices. Preoperative SII, as conventionally calculated from a CBC at a prespecified time point, is a candidate feature for Axis 1; serial SII may also be evaluated as one candidate trajectory. The mFI summarizes preoperative accumulated-deficit or frailty burden and predicts postoperative risk (7–9). Neither conventional implementation captures the full joint perturbation-response-resolution pattern or discordance among systemic inflammation, local joint status, neuromuscular recovery, and mobility. Incremental value must therefore be tested against time-matched SII, mFI, age, comorbidity, baseline pain, and function; it is not presumed.

Reproducibility is evaluated at the level appropriate to each construct. Repeat preoperative CBC or CRP, when clinically feasible, will quantify short-term reliability and reduce misclassification from transient illness. For Axes 2 and 3, expected time variation is the signal; robustness concerns the derived peak, AUC, slope, and recovery-time estimates under plausible sampling error. Exploratory class solutions require sensitivity analyses for starting values, model specification, and resampled datasets, together with posterior assignment uncertainty (29–32); similar patterns and assignment behavior must then be reproduced in an independent sample before labels are locked. Prediction models constructed from continuous or class-derived features additionally require assay alignment, site-effect assessment, protocol-specific calibration, and geographically independent validation (33).

## 4 Mechanistic bridge from inflammation to rehabilitation performance

### 4.1 Pain, swelling, and altered afferent input

Inflammatory mediators amplify nociceptive signaling and contribute to vascular permeability, effusion, and limb swelling. Serum IL-6, prostaglandin E2, CRP, and muscle-damage markers correlate with acute pain after TKA (34). The local response can then influence joint afferent input. Peripheral nociception at six weeks is associated with reduced voluntary activation and quadriceps weakness, with activation mediating part of the relationship (35). This sequence provides a plausible bridge from molecular exposure to a specific rehabilitation impairment.

Persistent pain adds synovial and central components. At six months, patients with moderate-to-severe pain showed more synovitis and effusion, greater temporal summation, and less efficient conditioned pain modulation than patients with lower pain (25). Long-term inflammatory mediator profiles also differ across pain, catastrophizing, and functional subgroups (24). These observations support multimodal phenotyping: an inflammatory signal gains meaning when paired with swelling, pain sensitivity, effusion, and activation.

### 4.2 Arthrogenic muscle inhibition and muscle capacity

Quadriceps weakness after TKA arises from both neural activation failure and loss of contractile capacity. Early work demonstrated that voluntary activation failure accounts for a large share of the acute strength loss (38), while preoperative quadriceps strength predicts later function (39). Arthrogenic muscle inhibition (AMI) therefore provides a mechanism-aligned endpoint for inflammatory phenotyping. A recent review identified compression, voluntary contractions, and neuromuscular electrical stimulation (NMES) as promising perioperative strategies for AMI (40).

Rehabilitation trials show that this pathway can be targeted. Early NMES improves quadriceps strength and function (41), and higher-intensity rehabilitation can be delivered safely after TKA (42). Mechanistic work indicates that NMES preserves muscle strength and fiber size during early recovery (43), while meta-analysis supports benefits across randomized studies (44). These data justify voluntary activation and quadriceps strength as proximal trial outcomes when testing candidate acute-response or swelling-dominant profiles.

Muscle capacity adds a second treatment target. Perioperative essential amino acid supplementation improved quadriceps strength and muscle-volume recovery in a double-blind randomized trial (45). Mitochondrial function, grip strength, and activity are also related to mobility recovery after TKA (46). The inflammatory phenotype can therefore be paired with a muscle-capacity profile, allowing NMES to address activation failure and progressive resistance exercise plus nutritional support to address contractile recovery.

### 4.3 Recovery trajectories as phenotype outputs

Pain and function after TKA follow distinct long-term trajectories (47). Model-based analyses identify outcome classes that are obscured by group averages (48), and latent-class analysis during the first six weeks demonstrates separable rehabilitation trajectories (37). These patterns are important because a dynamic inflammatory phenotype should predict an entire trajectory and capture more than a single endpoint. Repeated pain, strength, TUG, Knee injury and Osteoarthritis Outcome Score (KOOS), and activity measurements can reveal when recovery begins to diverge.

Multiomic endotyping provides a complementary discovery route. Deep learning-based clustering of knee osteoarthritis multiomic data has been used for endotyping and post-arthroplasty response classification (36). A systematic review of metabolic and inflammatory predictors similarly highlights the potential of combined biological signatures (49). The proposed three-axis model supplies a clinically interpretable scaffold for these data-rich approaches: molecular clusters can be tested for correspondence with trait, response, and resolution patterns.

## 5 Translating the phenotype into adaptive ERAS and rehabilitation

### 5.1 A tiered measurement strategy

A scalable program can use three tiers. The first tier combines preoperative CRP, complete blood count, body mass index, diabetes status, pain, and quadriceps strength. These measures are inexpensive and widely available. The second tier adds IL-6, serial CRP, swelling, and peri-patellar temperature during the first 72 h. The third tier adds targeted cytokine panels, synovial measures, fibrinogen-to-albumin ratio, effusion imaging, pain-sensitization testing, and wearable-derived mobility when the clinical or research question requires greater resolution.

For implementation, these tiers should be nested around a minimum viable dataset (MVD). The MVD uses routine preoperative CBC-derived ratios and CRP when already available, BMI or diabetes, activity pain, and simple function; one in-hospital or discharge checkpoint aligned with routine care; a week-2 phone or clinic review of wound, symptoms, pain, swelling, mobility, and adherence; and routine week-6 KOOS and TUG or an equivalent test. It does not require IL-6, continuous temperature, a wearable, or post-discharge research phlebotomy. Enhanced modules add denser 12–24- and 48–72-h sampling, serial IL-6 or CRP, AUC estimation, targeted cytokines, imaging, continuous temperature, and wearable mobility.

The resulting measurements should be summarized as continuous features. Peak, slope, AUC, and time-to-recovery retain information that is lost by early dichotomization. Institution-specific reference curves can account for ERAS components, surgical technique, tourniquet exposure, assay platform, and sampling schedule. A standardized core dataset then allows multicenter pooling while preserving local calibration. These trajectory features are available only when the enhanced schedule supports them; the MVD provides lower-resolution screening proxies. MVD and enhanced models should be prespecified, locked, and validated separately. No model-based prediction or class assignment should be issued unless the prespecified model supports the observed missing-data pattern.

### 5.2 Phenotype-matched anti-inflammatory treatment

Randomized trials demonstrate that perioperative glucocorticoids can alter inflammatory markers, pain, opioid consumption, and selected functional outcomes. Methylprednisolone markedly reduced CRP without improving early knee-extension strength or TUG in one trial (50). Dexamethasone improved early quadriceps power and walking in another study (51), and the DEX-2-TKA trial showed reduced morphine consumption and pain with repeat dosing (52). Trials conducted in different clinical pain-risk strata established the feasibility of stratified recruitment and produced differing results, but cross-trial contrasts do not constitute a formal treatment-by-stratum or biomarker interaction (53–55). Additional trials report early clinical benefits and, in some trials, functional benefits with low-dose or repeated corticosteroid regimens (56–59), while meta-analysis supports an overall perioperative effect profile (60).

However, the glucocorticoid evidence is heterogeneous in dose, timing, population, and endpoint. Trials have compared fixed 8- to 24-mg dexamethasone regimens, weight-based 0.3 vs. 1 mg/kg dosing, repeat postoperative dosing, methylprednisolone 125 mg, and multiple hydrocortisone schedules (50–60). Populations range from unselected TKA recipients to pain-risk strata and selected adults aged at least 75 years; biomarker ascertainment ranges from serial CRP or IL-6 to no mechanism-centered sampling, and outcomes range from analgesia and opioid use to early quadriceps function and TUG. Most individual trials had short follow-up and were not designed to exclude uncommon harms. Collectively, these trials demonstrate that the inflammatory response can be perturbed, but they do not establish that a biomarker-defined phenotype selects treatment benefit or that between-trial differences constitute interaction evidence.

These results offer a strong design opportunity. The biomarker should serve as a treatment-effect modifier, and the functional endpoint should match the proposed mechanism. Participants should be randomized across the prespecified continuous Axis 2 score distribution, with the score tested as an effect modifier of standard vs. protocol- or trial-based intensified anti-inflammatory treatment; IL-6/CRP AUC should assess target engagement, and voluntary activation, strength, TUG, pain during mobilization, and glucose safety should serve as downstream outcomes. This approach distinguishes biological suppression from meaningful recovery benefit. The intervention evidence, biological target engagement, functional findings, phenotype implications, and major cautions are compared in Table 3.

**Table 3:** Illustrative intervention evidence relevant to phenotype-adapted recovery after TKA (targeted, non-systematic synthesis).

Study (reference)	Design and analyzed sample	Population or stratum	Intervention and comparator	Biological target engagement	Functional or clinical result	Phenotype implication and key caution
Lindberg-Larsen et al. (50)	Randomized double-blind trial; 61 completers	Fast-track unilateral TKA	Methylprednisolone 125 mg IV before surgery vs. saline	CRP was markedly lower at 24 and 48 h	No improvement in knee-extension strength, swelling, TUG, or test-related pain at discharge	Biological suppression alone did not restore function; target engagement and mechanism-aligned function must be co-primary concepts
Chan et al. (51)	Three-arm randomized trial; 146 participants	Unselected primary TKA	Dexamethasone 8 mg, 16 mg, or placebo before surgery	Dose-response signal was assessed clinically; biomarker target engagement was not the central endpoint	The 16-mg group had less pain and opioid use, stronger early quadriceps power, and longer day-1 walking distance; no 3–12-month advantage	Suggests an early functional window, but no durable benefit and no biomarker-defined enrichment
Gasbjerg et al. (52)	Multicenter randomized trial; 472 in primary analysis	TKA under standardized multimodal analgesia	Dexamethasone 24 mg once, 24 mg repeated at 24 h, or placebo	Not biomarker-focused	Two doses reduced 0–48-h morphine use by a median 10.7 mg vs. placebo and reduced pain through 48 h	Defines a feasible dosing perturbation; clinical importance and responder heterogeneity still require testing
Nielsen et al. (53)	Randomized double-blind trial; 88 participants	High-pain-response stratum: catastrophizing score above 20 or regular opioid use	Dexamethasone 1 mg/kg vs. 0.3 mg/kg IV preoperatively	High dose reduced CRP at 24 and 48 h	Moderate-to-severe walking pain at 24 h was lower with high dose; quality of recovery improved	Supports enriched trials in a clinically defined high-risk stratum; metabolic and wound safety require adequate power
Nielsen et al. (54)	Two-center randomized double-blind trial; 157 analyzed	Low-pain-response stratum: low catastrophizing and no opioid use	Dexamethasone 1 mg/kg vs. 0.3 mg/kg IV	High dose lowered CRP at 48 h	No improvement in walking pain, opioid use, recovery quality, length of stay, or complications	Contrasting result with the high-pain stratum strengthens the rationale for treatment-effect heterogeneity testing, but it is not a formal biomarker interaction analysis
Springborg et al. (55)	Multicenter randomized trial; 110 participants	Persistent high pain at 24 h despite preoperative dexamethasone 1 mg/kg	Oral dexamethasone 24 mg on postoperative day 1 vs. placebo	Not reported as the primary mechanism endpoint	No statistically significant reduction in moderate-to-severe walking pain at 48 h or rescue analgesia	A second high dose after strong preoperative treatment may show limited incremental benefit; timing and ceiling effects matter
Abdelhameed et al. (56)	Randomized double-blind trial; 86 participants	Primary unilateral TKA	Dexamethasone 8 mg IV vs. placebo	CRP was lower at most early time points	Less pain, better TUG on days 1–2, and stronger quadriceps on days 2–3	Low-dose regimen is testable in stratified trials; small single-center safety dataset and short follow-up
Langkilde et al. (61)	Secondary analysis of a rehabilitation RCT; 60 participants had at least one preoperative and postoperative blood sample	TKA receiving rehabilitation with or without progressive strength training	Rehabilitation plus progressive strength training vs. rehabilitation alone	Serial IP-10, suPAR, IL-6, IL-10, and TNF-alpha through week 26	Baseline or surgery-induced inflammation did not modify the 6-minute-walk response; only IL-10 response associated with later change	Important null interaction. Sensitivity may have been limited by secondary analysis; 60 participants with paired perioperative sampling; individual biomarkers rather than a prespecified multivariate trajectory; one early postoperative sample, precluding individual peak, AUC, or decay estimates; a distal 26-week 6-minute-walk endpoint; and an intervention not primarily designed as anti-inflammatory. A true null remains possible.
Li et al. (57)	Three-arm randomized trial; 103 participants	Primary TKA	Saline, two 100-mg hydrocortisone doses, or four 100-mg doses	Four doses produced the greatest IL-6 and CRP suppression through 48 h	Multiple doses improved early pain, rehabilitation indicators, satisfaction, and length of stay	Supports dose-response experimentation, but findings require multicenter replication and contemporary safety assessment
Wu et al. (59)	Three-arm randomized trial; 150 participants	Primary TKA	Placebo, one 10-mg dexamethasone dose, or two 10-mg doses	One and two doses lowered CRP and IL-6; two doses had the strongest effect	Two doses improved pain, nausea/vomiting, range of motion, and satisfaction without observed complication differences	Provides a practical repeat-dose regimen; trial was not enriched by inflammatory phenotype
Xu et al. (62)	Prospective randomized controlled study; 60 analyzed (72 randomized)	Adults aged <70 years undergoing standardized primary TKA for osteoarthritis	Celecoxib 200 mg orally twice daily plus as-needed tramadol vs. as-needed tramadol alone for 6 weeks beginning postoperative day 1	Lower periarticular skin temperature at all postoperative assessments; lower CRP at weeks 1 and 6, IL-6 at week 1, and ESR at week 6; no WBC difference	Knee Society Score was higher at week 1, with no significant group difference at weeks 2 or 6	Supports a non-glucocorticoid perturbation of systemic-local inflammatory resolution; single-center selected cohort, 12/72 attrition, 6-week follow-up, and no biomarker-defined treatment interaction limit inference
Xu et al. (58)	Randomized double-blind trial; 153 completers	Adults aged 75 years or older with ASA physical status I-II	Dexamethasone 10 mg IV vs. saline	IL-6 and CRP were lower with dexamethasone	Lower 24-h pain and nausea/vomiting; no increase in observed infection or delayed wound healing	Demonstrates feasibility in selected older adults; extrapolation to frail or highly comorbid patients is uncertain
Ueyama et al. (45)	Double-blind randomized trial; 52 analyzed	Primary unilateral TKA	Essential amino acids 9 g/day from 1 week before to 2 weeks after surgery vs. placebo	Muscle preservation rather than inflammatory suppression was targeted	Greater rectus femoris area and quadriceps strength at 2 years; no significant clinical-outcome difference	Supports a parallel catabolic/low-capacity phenotype, but muscle gains did not translate into measured global clinical benefit
Quesnot et al. (63)	Randomized trial; 40 participants	Post-TKA rehabilitation	Compressive cryotherapy vs. standard cryotherapy	Greater reduction in effusion and faster reduction in knee circumference	Better activity pain, 6-minute walk, KOOS, and faster range-of-motion recovery by day 21	Provides a local-response intervention for swelling-dominant phenotypes; small sample and short follow-up
Liu et al. (60)	Systematic review and meta-analysis; 36 randomized trials	Perioperative TKA populations	Glucocorticoids vs. control; systemic and periarticular approaches	Lower postoperative inflammatory cytokines	Small short-term improvements in pain, opioid use, range of motion, nausea/vomiting, and length of stay	Overall efficacy is short-term and heterogeneous; postoperative day-1 glucose increases require explicit metabolic safety monitoring

### 5.3 Phenotype-matched rehabilitation

The rehabilitation response should address the dominant impairment. Candidate swelling-dominant and activation-failure profiles provide hypotheses for prospective testing of compression, cryotherapy, NMES, and frequent activation practice; assignment to any adaptation should occur only within a protocol or trial after safety triage. Compressive cryotherapy has been evaluated against standard cryotherapy for pain, swelling, range of motion, and function (63). High preoperative inflammatory-tone with low baseline strength and delayed-resolution profiles may motivate prospectively tested nutrition-resistance or prolonged supervised-rehabilitation strategies, respectively, but these remain research hypotheses rather than recommendations for routine care.

Multimodal analgesia remains the common foundation. Procedure-specific recommendations support a coordinated regimen that enables early mobilization (64). Candidate-profile adaptation is a prospective research strategy that retains the standardized core ERAS pathway while testing the intensity, timing, and monitoring of selected components. Some arthroplasty programs already use compression, NMES, targeted exercise, laboratory testing, and follow-up contacts; where these resources are unavailable, implementation should prioritize the MVD, non-device alternatives, phone follow-up, and clinically indicated local or home sampling rather than make access to equipment a condition of care.

### 5.4 Adaptive checkpoints across the recovery pathway

The framework can be embedded at four clinical checkpoints. The preoperative checkpoint establishes inflammatory tone, strength, pain, metabolic risk, and expectations. It creates the initial recovery profile and identifies patients who need intensified preparation. The in-hospital checkpoint combines the early biomarker trajectory with swelling, pain during movement, and initial quadriceps activation. It supports timely adjustment of anti-inflammatory management, compression, activation practice, and discharge planning.

At the early post-discharge checkpoint, a lower-burden MVD uses a phone or clinic review of wound status, symptoms, activity pain, swelling, mobility, and adherence; temperature or wearable streams are optional enhanced modules. The six-week checkpoint uses routine symptom and functional assessment to determine whether recovery is aligned with protocol-specific expectations. At every checkpoint, routine complication surveillance runs in parallel and takes priority: red flags, atypical combinations, or clinician concern suspend candidate-pattern-directed escalation and trigger standard diagnostic evaluation. Candidate-pattern review may proceed only after urgent causes are excluded, or after indicated treatment or referral, clinical stabilization, and a repeat safety screen permits re-entry.

**Figure 2:**
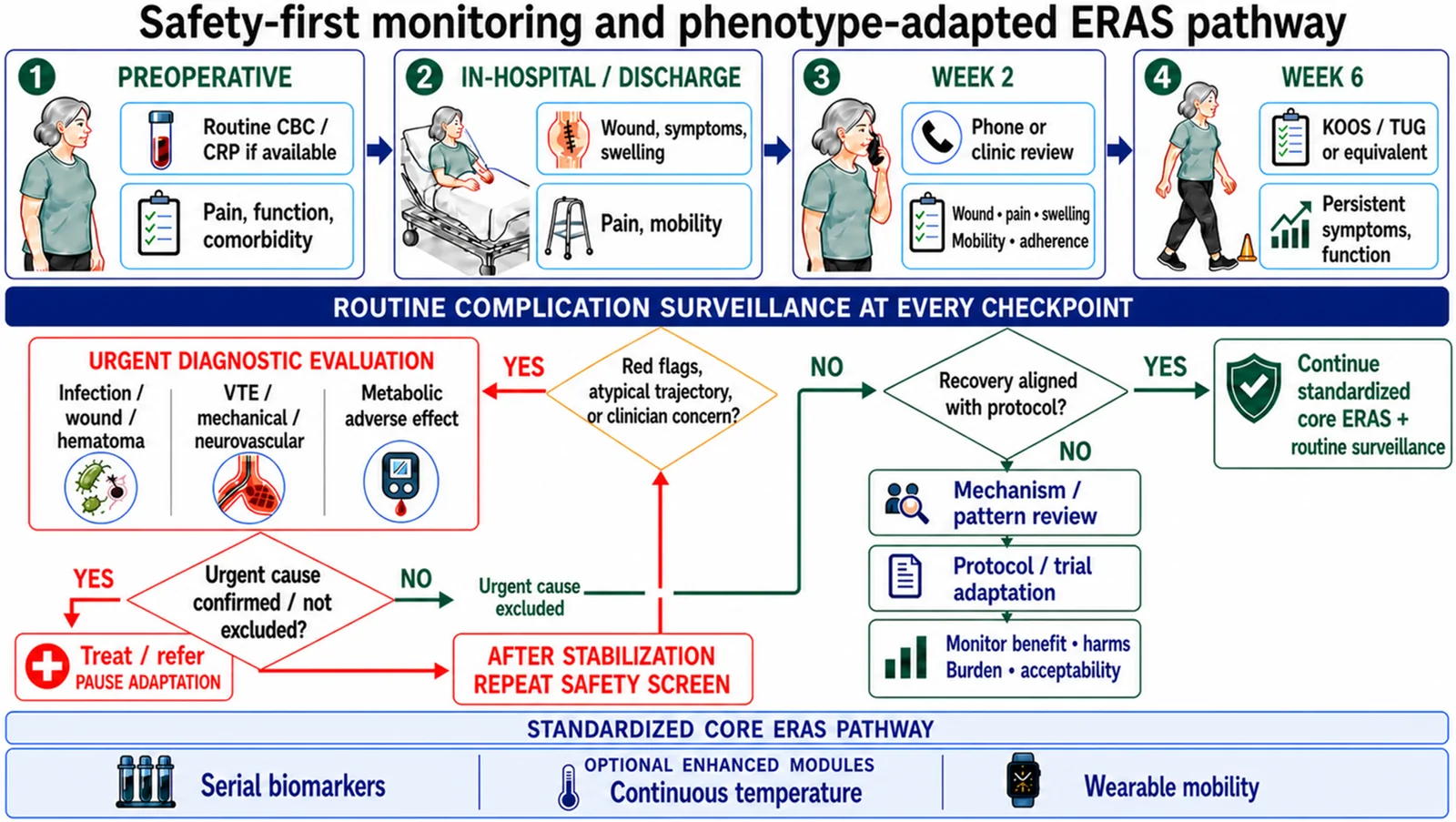
Safety-first monitoring and phenotype-adapted ERAS pathway after total knee arthroplasty. The minimum viable pathway uses routine preoperative data, one in-hospital or discharge assessment, a week-2 phone or clinic review, and routine week-6 functional assessment; serial IL-6 or CRP, continuous peri-patellar temperature, and wearable mobility are optional enhanced modules. Routine complication surveillance applies to every patient at every checkpoint and takes priority over candidate-pattern adaptation. Red flags, an atypical trajectory, or clinician concern trigger clinician-led evaluation for infection, wound complication or hematoma, venous thromboembolism, mechanical or neurovascular problems, and treatment-related metabolic adverse effects. If an urgent cause is confirmed or cannot safely be excluded, candidate-pattern adaptation remains paused while indicated treatment or referral proceeds. If an urgent cause is excluded, recovery alignment is assessed. Following indicated treatment or referral and clinical stabilization, the safety screen is repeated before any re-entry to recovery-alignment review. If recovery remains misaligned with protocol-specific expectations, a mechanism or candidate-pattern review may support protocol- or trial-based adaptation, followed by monitoring of benefit, adverse effects, burden, and acceptability. No single biomarker, temperature value, swelling measure, or device alert should independently rule in or rule out a complication or trigger treatment. The framework is proposed for prospective validation and is not a validated diagnostic or treatment algorithm. ERAS, enhanced recovery after surgery; IL-6, interleukin-6; CRP, C-reactive protein; KOOS, Knee injury and Osteoarthritis Outcome Score; TUG, Timed Up and Go.

This checkpoint structure gives nurses, physiotherapists, surgeons, and anesthesiology teams defined roles. Nurses can coordinate serial measurements, wound and glucose surveillance, symptom review, and remote alerts. Physiotherapists can quantify activation, strength, mobility, and training response. Surgeons can evaluate mechanical or complication-related explanations for an atypical trajectory, while perioperative physicians can tailor anti-inflammatory and analgesic strategies. Shared data at predefined checkpoints reduce fragmented escalation and make prospective pathway evaluation operational within multidisciplinary care. The proposed checkpoint-based adaptive pathway is summarized in Figure 2, and the corresponding phenotype-linked decisions, mechanism outcomes, clinical outcomes, and safety gates are specified in Table 4. No device alert should diagnose a complication or trigger treatment without clinician review.

**Table 4:** Provisional recovery patterns and candidate adaptations after safety triage for prospective testing.

Provisional recovery pattern	Decision checkpoint	Minimum confirmation	Escalated characterization	Candidate adaptation	Proximal mechanism outcome	Clinical outcome	Safety gate and stop rule
High preoperative inflammatory tone with metabolic risk	Preoperative assessment	Repeat CRP/CBC when clinically appropriate; BMI, diabetes status, pain, and quadriceps strength	Targeted cytokines or synovial measures; nutrition and frailty assessment	Metabolic optimization, nutrition and strength preparation, intensified perioperative monitoring, and trial-based anti-inflammatory adaptation	Change in IL-6/CRP response; glucose trajectory; activation and strength preservation	Mobilization, TUG, KOOS, length of stay, and complications	Exclude active infection or uncontrolled inflammatory disease; monitor hyperglycemia and wound risk; do not treat a single elevated marker in isolation
Exaggerated acute systemic response	12–72 h after surgery	IL-6/CRP deviation from a protocol-specific curve plus pain during movement and initial mobility	Peak, slope, and AUC; review operative stressors, medication exposure, and local findings	Pattern-guided anti-inflammatory dosing within a trial or approved protocol; reinforce multimodal analgesia and activation practice	IL-6/CRP AUC, pain during activity, opioid use, and voluntary activation	Walking distance, TUG, discharge readiness, and early KOOS	Structured assessment for infection, hematoma, thromboembolism, or other complication; monitor glucose and wound status; stop escalation if the clinical pattern is atypical
Swelling-dominant local response with activation failure	In hospital through week 2	Standardized swelling or effusion measure plus voluntary activation or quadriceps strength	Bioimpedance or ultrasound; pressure-pain threshold; gait and TUG	Compression or compressive cryotherapy, NMES, frequent activation practice, and individualized load progression	Peak swelling, effusion, activation, quadriceps strength, and activity pain	Gait speed, TUG, stairs, 6-minute walk, and KOOS	Check skin integrity, neurovascular status, wound drainage, suspected thrombosis, and device tolerance; suspend compression if adverse local signs emerge
Catabolic or low-capacity profile	Preoperative to week 6	Baseline strength, functional mobility, BMI, dietary screening, and relevant routine laboratories	Muscle ultrasound, activity exposure, and selected metabolic or mitochondrial measures	Protein or essential amino acid support when suitable plus progressive resistance training	Muscle thickness or area, strength, activation, and achieved training dose	KOOS function, mobility, fatigue, and participation	Confirm renal, hepatic, nutritional, and swallowing suitability; monitor exercise intolerance and avoid assuming muscle gain guarantees global functional benefit
Delayed resolution pattern	Week 2 and week 6	MVD: swelling or effusion, activity pain, activation or simple function, and TUG or KOOS at routine checkpoints. Enhanced: repeat CRP or a fibrinogen-based marker when clinically obtained or prespecified.	Imaging for persistent effusion or synovitis; exploratory thermo-mobility coupling indices; sensitization and psychosocial assessment	Extended supervised rehabilitation, repeated mechanism assessment, and targeted local, neuromuscular, nutritional, or pain intervention	Decay slope, residual inflammation, effusion, activation, strength, and trajectory re-entry	KOOS, TUG, persistent pain, activity recovery, and return to participation	First evaluate wound complication, infection, thromboembolism, mechanical problems, or hematoma. Candidate-pattern adaptation may proceed only after urgent causes are excluded, or after indicated treatment or referral, clinical stabilization, and a repeat safety screen permits re-entry.
Discordant remote recovery pattern	Early post-discharge through week 6	Wound or symptom check plus patient-reported, telephone, clinic, temperature, or mobility trend; wearable not required.	Exploratory continuous thermo-mobility coupling, adherence review, and targeted in-person examination	Triggered remote contact, rapid clinical review, and rehabilitation dose adjustment based on the identified mechanism	Candidate thermo-mobility coupling indices, step recovery, pain-activity relationship, and adherence	Earlier recognition of stalled recovery, KOOS, TUG, and unplanned care	Use validated alert logic, protect privacy, confirm device adherence, and require clinician review rather than automated treatment escalation

Patterns and pathways are non-exclusive. Routine complication surveillance continues at every checkpoint. Atypical or red-flag patterns require clinician-led diagnostic evaluation, and candidate-pattern review or adaptation may proceed only after urgent causes are excluded, or after indicated treatment or referral, clinical stabilization, and a repeat safety screen permits re-entry. No single biomarker, temperature value, swelling measure, or device alert should independently diagnose, rule out, or trigger treatment.

## 6 Testable predictions

### 6.1 Prediction 1: the three-axis model will provide incremental value beyond isolated markers and conventionally implemented indices

Incremental value must be evaluated at prespecified prediction landmarks. A preoperative model can include Axis 1 and predict subsequent early activation, swelling, mobility, or pain outcomes. A 24–72-h model may add Axis 2 information available by that landmark to predict later discharge or post-discharge outcomes. Axis 3 resolution information becomes available only at later checkpoints, such as week 2, and may predict week-6 or later outcomes; it must not be used to predict earlier or concurrent endpoints. Time-matched SII and preoperative mFI should be included as explicit reference models. Incremental performance is a prospective hypothesis, not an established advantage.

### 6.2 Prediction 2: phenotype-by-treatment interactions will be outcome specific

Patients with an exaggerated acute response should show greater biological target engagement from intensified anti-inflammatory treatment. Functional benefit should be largest when inflammation or swelling is a dominant limiter of movement. The same continuous response profile or score may have less influence on outcomes driven primarily by central sensitization, severe preoperative weakness, anemia, sleep disruption, or social barriers. This prediction favors a panel of mechanism-aligned endpoints over a single global recovery score.

### 6.3 Prediction 3: local and systemic measures will provide complementary information

Systemic IL-6 and CRP should capture the whole-body surgical response, while swelling, effusion, and temperature should capture the local recovery environment. Discordance between these signals is expected. A patient may show a typical CRP trajectory and persistent local swelling, or an elevated systemic response with limited local dysfunction. Combining both levels should improve phenotype specificity and guide the choice between systemic treatment, local swelling management, and rehabilitation intensification.

### 6.4 Prediction 4: early neuromuscular endpoints will reveal treatment effects before global function

Voluntary activation and quadriceps strength should respond earlier than broad patient-reported outcomes. A phenotype-adapted intervention may first improve activation, reduce swelling, or increase walking dose, followed by later improvement in TUG and KOOS. Serial measurement can establish this sequence and strengthen causal interpretation through mediation analysis.

### 6.5 Prediction 5: dynamic updating will improve clinical utility

The preoperative axis profile should provide an initial probability of difficult recovery. Acute biomarker and swelling data can update that probability during the first 72 h, and temperature, mobility, pain, and function can update it again during weeks 2–6. A dynamic model should therefore be tested against a time-matched preoperative reference model to determine whether it improves prediction and detects recoveries that change direction after discharge; outperformance is a prospective hypothesis, not an assumption.

## 7 Validation pathway

The first stage is a multicenter prospective cohort with harmonized sampling. For enhanced validation cohorts, a prespecified research schedule may include a preoperative baseline, 24 h, 48–72 h, 2 weeks, and 6 weeks. Daily swelling or temperature during the first postoperative week and wearable mobility during weeks 1–6 are optional enhanced modules rather than requirements of the MVD; lower-resource implementation can use the routine-care checkpoints defined in Table 2. Core outcomes should include pain during activity, opioid use, voluntary activation, quadriceps strength, TUG, gait speed, KOOS domains, and complications.

Phenotype derivation should prioritize prespecified continuous features. Latent-class or trajectory clustering is an exploratory secondary representation and should be retained only if the solution is adequately separated, substantively interpretable, robust to starting values and model specification, stable across resampled datasets with sensitivity analyses reported transparently, and reproduced in an independent sample (29–32). For prediction-model development, all preprocessing, imputation, continuous feature construction, and predictor selection should be completed within the resampling loop. Exploratory mixture modeling should separately report enumeration criteria, convergence, starting-value checks, posterior classification uncertainty, and sensitivity to model specification and resampled datasets; the selected solution requires independent-sample replication before its labels are locked. Prediction transformations and rules should then be tested in a geographically separate cohort. Calibration, discrimination, and action-specific decision-curve analysis should be reported.

Incremental comparisons should be landmark based. A preoperative model should compare Axis 1 with preoperative SII, mFI, and conventional variables; a 24–72-h model may add only Axis 2 information available by that landmark; and a week-2 model may add resolution information observed by week 2. Each model should predict outcomes occurring after its landmark, with no future measurements entering predictor construction. A swelling, activation, TUG, or other functional measure used as a predictor at a landmark should not also serve as the same-time outcome; it should predict a later endpoint to avoid incorporation bias.

For continuous outcomes, sample-size calculations should use the anticipated outcome R-squared and the total candidate parameter degrees of freedom, including nonlinear terms and interactions, following Riley et al. Part I (65). For binary or time-to-event outcomes, calculations should use the anticipated event proportion and the corresponding criteria in Riley et al. Part II (66). Both calculations should target acceptable shrinkage and optimism and allow for expected unusable or missing observations rather than apply a fixed events-per-variable rule. For exploratory latent or trajectory models, Monte Carlo simulations tailored to the intended longitudinal mixture model should vary the expected number of classes, smallest-class prevalence, separation, number of repeated measures, and missingness (30–32). Candidate classes with very small, unstable, or site-specific membership should not be promoted to clinical phenotypes.

Overfitting control requires more than a final bootstrap correction. The full prediction pipeline, including harmonization, imputation, feature extraction, and variable reduction, should be repeated within bootstrap or nested resampling; shrinkage or penalization should be applied when appropriate, and simple random split-sample development should be avoided. A locked model then requires geographic external validation, with calibration slope, discrimination, net benefit, and site heterogeneity reported for each outcome. Internal-external cross-validation can quantify between-site transportability before final external testing (33, 67). External validation of a class-based predictor additionally depends on the separate mixture-model checks and independent replication described above; downstream bootstrap correction does not validate the class solution. For external validation of binary-outcome models, sample size should be planned for sufficiently precise estimates of calibration and discrimination rather than selected by convenience (68). Continuous-outcome external validation should be planned separately with outcome-appropriate precision targets.

Decision-curve thresholds must be defined by the action under consideration, not by a biomarker cut point or the value that maximizes apparent performance. Each threshold range must specify the prediction landmark, outcome, prediction horizon, and clinical action. A threshold probability is the predicted risk at which the expected benefit of acting is judged to offset the harm and burden of unnecessary escalation. Prespecified ranges should be elicited from patient and clinician preferences, treatment harms, resource requirements, and capacity: a low-burden remote review may justify a lower threshold than systemic glucocorticoid intensification or resource-intensive rehabilitation. Net benefit should be compared with treat-all and treat-none strategies across those action-specific ranges, without *post hoc* threshold selection (69, 70).

Clinical utility requires more than statistical separation. The phenotype should improve calibration and net benefit after conventional predictors are included. Only a small set of mechanistically justified effect modifiers should be prespecified, potentially including age, sex, adiposity, diabetes, baseline pain sensitization, anesthetic technique, surgical technique, tourniquet use, or ERAS protocol. Formal interaction estimates and confidence intervals should be reported; exploratory analyses should address multiplicity and limited power, and heterogeneity should not be inferred merely because one subgroup is statistically significant and another is not. These analyses can examine whether the same inflammatory trajectory carries comparable meaning across selected clinical contexts and where local recalibration may be required.

The second stage is a biomarker-stratified randomized trial. Participants can be randomized with prespecified continuous axis scores used as effect modifiers; if externally reproduced and locked cut points exist, pragmatic trial strata may also be used to compare standard with adapted anti-inflammatory care and standard with AMI-focused rehabilitation. A factorial design can estimate the independent and combined effects of these adaptations. The primary analysis should test treatment-by-phenotype interaction, supported by the Predictive Approaches to Treatment effect Heterogeneity framework (71). This strategy aligns with the PROGRESS recommendations for stratified medicine research by separating prognostic enrichment from prediction of differential treatment benefit (72). Any trial strata are pragmatic enrollment and analysis tools, not assumed biological classes.

A previous secondary trial analysis directly tested baseline and surgery-induced inflammation as modifiers of progressive strength training and reported a null interaction for change in six-minute walking distance (61). That study provides a valuable design anchor for the next generation of trials. Interaction power should be planned prospectively, the phenotype should be defined against a reference trajectory, and proximal quadriceps activation or strength outcomes should accompany global walking measures.

Interpretation of that null interaction should remain balanced. The analysis was secondary, and 60 participants had at least one preoperative and postoperative blood sample. Individual biomarkers were evaluated rather than a prespecified multivariate trajectory construct. The single postoperative-day-1 sample may have been near an early peak but could not estimate individual peak timing, AUC, or early decay slope; later samples occurred at weeks 4, 8, and 26. Progressive strength training was not primarily designed as an anti-inflammatory perturbation, and 26-week six-minute walking distance is a distal, multifactorial endpoint that may be less sensitive than early activation or quadriceps strength. These design features may reduce interaction sensitivity, and interaction tests generally require larger samples (73), but a true absence of effect modification remains a plausible interpretation.

The interaction-trial sample-size calculation should be based on the interaction effect and account for the distribution of the continuous effect modifier or the prevalence of prespecified trial strata. Target engagement should be established through biomarker AUC, swelling, activation, and strength. Clinical relevance should then be tested through TUG, KOOS, and mobility at 6–12 weeks, with persistent pain and participation assessed later. Mediation analysis can determine whether changes in inflammation or swelling precede neuromuscular and functional benefit. This interaction-trial calculation is distinct from the phenotype-derivation calculations described above.

## 8 Implementation requirements and safety gates

Clinical translation requires a safety-first separation between routine complication surveillance and recovery phenotyping. Routine surveillance continues at every checkpoint. Red flags, an atypical trajectory, or clinician concern, including overlapping signals such as elevated CRP with swelling, should suspend phenotype-directed escalation and trigger standard clinician-led evaluation for infection, wound complication or hematoma, venous thromboembolism, mechanical or neurovascular problems, and treatment-related metabolic adverse effects. Candidate-pattern review or adaptation may proceed only after urgent causes are excluded, or after indicated treatment or referral, clinical stabilization, and a repeat safety screen permits re-entry. Phenotype monitoring must not replace or delay usual diagnostic care, and no single biomarker, temperature value, swelling measure, or device alert should independently rule in, rule out, or trigger treatment. Safety outcomes include glucose disturbance, wound healing, infection, thromboembolic events, neurovascular status, and treatment tolerance.

Feasibility endpoints should include recruitment and refusal reasons, checkpoint completion, extra phlebotomy and visits, staff time and cost, device activation, valid wear time, technical-support needs, withdrawal, and patient-reported burden. Research blood draws should be combined with clinically required phlebotomy whenever possible; post-discharge research sampling is optional and can be nested or collected locally or at home. Local or home collection should be used only with prespecified and validated procedures for collection tubes, processing delay, transport temperature, storage, and assay harmonization.

Digital measurements require a prespecified data-quality plan covering device model and firmware, placement, sampling frequency, wear-time and valid-day rules, calibration, charging and synchronization failures, non-wear detection, skin tolerance, slow gait and walking-aid performance, and reasons for missingness (74, 75). In one TKA tracking study, only 20 of 62 enrollees both underwent surgery and contributed device data, and average data retention differed by 24 percentage points between the most lenient and stringent valid-day criteria (74). Missingness may be informative in patients with pain, frailty, or cognitive or dexterity limitations and should be described by mechanism, with sensitivity analyses for non-random missingness. Wearable or temperature alerts require clinician confirmation and may not automatically trigger diagnosis or treatment.

Equitable implementation requires loan devices, connectivity and training support, caregiver assistance when appropriate, and telephone, paper, or clinic-based alternatives for patients without suitable devices or smartphones. Stated willingness is not equivalent to sustained adherence: in one survey, 83.6% of respondents were willing to wear a monitoring device, but willingness was lower among the oldest participants (76). Prospective validation should use fit-for-purpose procedures for training, technical support, and device and connectivity access (77). The MVD must remain usable without a wearable or post-discharge blood draw. Home-based, remote, or local rehabilitation alternatives should be available when specialized services are inaccessible, and actual rehabilitation dose should be recorded. Recruitment, completeness, calibration, discrimination, and net benefit should be reported across age, comorbidity, digital access, socioeconomic, and geographic groups where sample size permits, with uncertainty intervals.

## 9 Discussion

This framework contributes a decision-oriented organization of the TKA inflammation literature. Previous studies establish reference trajectories, prognostic associations, pharmacological modifiability, swelling-function relationships, and heterogeneous recovery classes. The three-axis model links these findings across the full perioperative course. It also clarifies where each measurement belongs: baseline markers describe susceptibility, early trajectories quantify the surgical response, and systemic-local recovery patterns describe resolution. Its proposed novelty lies in the temporal perturbation-response-resolution structure; incremental value over SII and mFI remains unproven.

The approach has several practical advantages. It begins with routine data, adds specialized markers when they increase information, and pairs every biological signal with a mechanism-aligned functional measure. It preserves the reliability of standardized ERAS while creating explicit points for adaptation. It also accommodates molecular, clinical, and digital data in a shared structure, which makes it suitable for both resource-conscious implementation studies and multiomic discovery cohorts.

The evidence base also defines the validation agenda. Existing cohorts vary in sampling schedules, assays, perioperative protocols, and outcomes. Several mechanistic studies are small, and current corticosteroid trials were designed mainly around analgesia. These features make external validation and interaction-powered trials essential. Concentrating these requirements in the next study design strengthens the framework: each uncertainty becomes a measurable prediction, a prespecified endpoint, or a safety gate.

This article is a targeted, non-systematic hypothesis synthesis; citation selection is not exhaustive, no pooled effects or formal risk-of-bias judgments are offered, and treatment evidence should not be read as a comparative efficacy review. Glucocorticoid heterogeneity, sparse sampling, small mechanistic cohorts, device-related missingness, and access constraints may all limit transportability. The continuous three-axis representation, any exploratory class solution, and the lower-burden MVD therefore require separate prospective validation.

## 10 Conclusion

Perioperative inflammation provides a measurable, modifiable, and mechanistically coherent basis for precision recovery after TKA. A three-axis phenotype spanning preoperative inflammatory tone, acute response magnitude, and resolution kinetics can connect molecular signals with pain, swelling, quadriceps activation, mobility, and long-term recovery trajectories. The framework supports a tiered measurement strategy and defines phenotype-adapted hypotheses for anti-inflammatory treatment, compression, NMES, nutritional support, progressive resistance exercise, and extended rehabilitation. Multicenter trajectory validation followed by interaction-powered randomized trials can establish clinical utility. This program offers a direct route from standardized ERAS to adaptive, biomarker-stratified recovery care. Until such validation, the framework should be regarded as a hypothesis-generating, safety-gated decision structure rather than a clinical classification or treatment algorithm.

## Data Availability

No new datasets were generated or analyzed for this article. The evidence discussed is available in the cited publications.
